# The Effect of Sufentanil Administration on Remifentanil-Based Anaesthesia during Laparoscopic Gynaecological Surgery: A Double-Blind Randomized Controlled Trial

**DOI:** 10.1155/2014/701329

**Published:** 2014-05-13

**Authors:** Ilsoon Son, Chung-Sik Oh, Jae Won Choi, Seong-Hyop Kim

**Affiliations:** ^1^Department of Anaesthesiology and Pain Medicine, Konkuk University Medical Centre, Konkuk University School of Medicine, 120-1 Neungdong-ro, Hwayang-dong, Gwangjin-gu, Seoul 143-729, Republic of Korea; ^2^Institute of Biomedical Science and Technology, Konkuk University School of Medicine, Seoul 143-701, Republic of Korea

## Abstract

This study assessed the effect of sufentanil administered before conclusion of remifentanil-based anaesthesia on postoperative hyperalgesia and haemodynamic stability in patients undergoing laparoscopic gynaecological surgery. The patients were randomly allocated to a sufentanil administration group (S group) or a normal saline administration group (C group). Anaesthesia was induced and maintained with controlled administration of remifentanil at 10 ng*·*mL^−1^ and propofol under bispectral index guidance. Once the surgical specimen was procured, sufentanil or normal saline was administered at 0.15 ng*·*mL^−1^ and maintained until extubation. The haemodynamic status during anaesthetic emergence was evaluated. The pain and postoperative nausea and vomiting (PONV) were assessed for 72 h following postanaesthetic care unit (PACU) discharge. The S group had significantly lower mean systemic arterial blood pressure and heart rate changes between the start of drug administration and extubation. Postoperative pain was significantly lower in the S group until 24 h following PACU discharge. There were no significant differences in PONV incidence and severity 72 h after PACU discharge between the two groups. Sufentanil administration before concluding remifentanil-based anaesthesia improved postoperative hyperalgesia and achieved haemodynamic stability at extubation without delaying recovery or increasing PONV during laparoscopic gynaecological surgery. Clinical trial registration is found at KCT0000785.

## 1. Introduction

The combination of propofol as a hypnotic agent and remifentanil as an analgesic agent is the most popular regimen for achieving stable haemodynamic and surgical states during total intravenous anaesthesia (TIVA) [1–3]. Generally, the required propofol dose is adjusted to maintain the bispectral index (BIS) between 40 and 60 during general anaesthesia [4], and the required remifentanil dose is adjusted maximally to mitigate the neurohumoral response to surgical stress during TIVA. Remifentanil is rapidly metabolized by unspecific blood and tissue esterases and the metabolites are largely inert [5]. Therefore, a patient administered a high intraoperative remifentanil dose may experience increased postoperative pain requiring additional analgesic agents immediately following remifentanil cessation [6, 7]. Patient anxiety and haemodynamic instability can occur during the postoperative period.

Sufentanil remains metabolically active longer than remifentanil [8], but sufentanil administration for a short duration results in early recovery [9]. Sufentanil administration during emergence from desflurane general anaesthesia reduced the postoperative analgesic requirement without increasing postoperative nausea and vomiting (PONV) [10]. Therefore, we hypothesized that sufentanil administration before anaesthetic conclusion may prevent postoperative hyperalgesia and haemodynamic instability during remifentanil-based anaesthesia. The present study assessed the effect of sufentanil administered before the conclusion of anaesthesia on postoperative hyperalgesia and haemodynamic parameters during laparoscopic gynaecological surgery under remifentanil-based anaesthesia.

## 2. Materials and Methods

### 2.1. Study Population

This prospective, double-blind, and randomised study was approved by the Institutional Review Board (KUH1160057, Institutional Review Board of Konkuk University Medical Centre, Seoul, Republic of Korea) and registered at http://cris.nih.go.kr (KCT0000785). Written informed consent was obtained from all patients. Patients undergoing laparoscopic gynaecological surgery with postoperative intravenous patient controlled analgesia (PCA) were enrolled. The exclusion criteria were as follows: (1) urgent or emergent case, (2) repeat procedure, (3) egg or soybean oil allergy, (4) drug abuse history, (5) current medications for 3 months which could influence postoperative pain and PONV, (6) prolonged QT on preoperative electrocardiography, (7) other concurrent surgeries, (8) surgical duration less than 1 h, (9) hospital discharge within 72 h, and (10) inability to be interviewed. The patients were randomly allocated to the sufentanil group (S group) or normal saline group (C group) using sealed envelopes containing the allocation. Participating anaesthesiologists, surgeons, and nurses were blinded to the study. All data were collected by trained observers who were blinded to the study and did not participate in patient care.

### 2.2. Anaesthetic Protocol

Preanaesthetic medication was not administered to the patients. Upon arrival to the surgical suite, routine patient monitoring was established, and anaesthesia was induced. The anaesthetic technique was standardized for both groups; lidocaine 0.5 mg·kg^−1^ was administered intravenously to decrease pain induced by propofol. An initial target concentration (effect-site, modified Marsh model with *k*_*e*0_ 1.21 min^−1^ [11]) of propofol 4 *μ*g·mL^−1^ and the fixed target concentration (plasma, Minto model [12, 13]) of remifentanil 10 ng·mL^−1^ were administered intravenously using two target controlled infusion (TCI) devices. The target remifentanil concentration of 10 ng·mL^−1^ was achieved 10 min after administration and maintained during anaesthesia. An initial target propofol concentration was titrated with 0.1 *μ*g·mL^−1^ increments to maintain the BIS between 40 and 60. Rocuronium 0.6 mg·kg^−1^ was administered intravenously to induce muscle relaxation after loss of consciousness, guided by peripheral neuromuscular transmission (NMT) monitoring. Endotracheal intubation was performed once the target concentration of remifentanil 10 ng·mL^−1^ was reached and the train-of-four count was 0. Additional rocuronium was administered under peripheral NMT monitoring. Once the surgical specimen was procured, sufentanil (S group) or normal saline (C group) was administered intravenously at a targeted concentration of 0.15 ng·mL^−1^ (plasma, Gepts' model) [9]. A 50 mL syringe containing 5 mL sufentanil (250 mg) and 45 mL normal saline (S group) or only 50 mL normal saline (C group) for TCI was prepared by a registered nurse blinded to the study and not participating in patient care.

The patient was intravenously administered 30 *μ*g phenylephrine (mean systemic arterial blood pressure [MBP] < 60 mmHg and heart rate [HR] > 40 beats·min^−1^), 4 mg ephedrine (MBP < 60 mmHg and HR < 40 beats·min^−1^), or atropine (HR < 40 beats·min^−1^), as needed, to prevent hypotension or bradycardia. Phenylephrine was continuously infused if the MBP < 60 mmHg persisted despite phenylephrine therapy. Nicardipine (0.5 mg) was intravenously administered at a systolic blood pressure > 180 mmHg or diastolic blood pressure > 110 mmHg, and 30 mg esmolol was administered intravenously at MBP > 60 mmHg and HR > 110 beats·min^−1^ during anaesthesia after the target remifentanil concentration was achieved. The remifentanil and propofol TCIs were stopped postoperatively after incision bandaging. Ketorolac (0.5 mg·kg^−1^) was administered intravenously to control postoperative pain, and an intravenous PCA pump was connected to the patient at surgery conclusion. The PCA regimen consisted of 1,500 *μ*g of fentanyl in normal saline to a total 150 mL volume administered only at basal dose of 0.02 mL·kg^−1^·h^−1^ without on-demand dose. Residual neuromuscular paralysis was antagonized with intravenous administration of 0.05-mg·kg^−1^ neostigmine and 0.01-mg·kg^−1^ glycopyrrolate under peripheral NMT monitoring. After endotracheal extubation, the administration of sufentanil (S group) or normal saline (C group) was discontinued, and the patient was transferred to the postanaesthetic care unit (PACU).

### 2.3. Measurements

The MBP, HR, and BIS were measured at sufentanil (S group) or normal saline (C group) initiation (*T*_*s*_) and after extubation (*T*_*e*_). The change in MBP (ΔMBP), HR (ΔHR), and BIS (ΔBIS) between *T*_*s*_ and *T*_*e*_ was calculated. The concentration at extubation, total infused amount, and infusion duration of sufentanil (S group) or normal saline (C group) were recorded. Anaesthetic and surgical durations and the emergence time were also recorded. The total infused remifentanil, propofol, phenylephrine, ephedrine, and atropine doses were also recorded.

Postoperative pain was assessed using the visual analogue scale (VAS, ranging from 0 to 100 mm: 0 = no pain and 100 = worst pain imaginable) on PACU arrival (*T*1), 30 min after PACU arrival (*T*2), and at 24 (*T*3), 48 (*T*4), and 72 h after PACU discharge (*T*5). Ketorolac (0.5 mg·kg^−1^) was administered intravenously as the first-line analgesic on demand. If ketorolac was not effective, then 0.2-mg·kg^−1^ meperidine was administered intravenously as the second-line analgesic on demand.

Postoperative nausea and vomiting (PONV) was assessed on a 3-point ordinal scale (0 = none, 1 = nausea, 2 = retching, and 3 = vomiting) [14] at *T*1 and between *T*1 and *T*2, *T*2 and *T*3, *T*3 and *T*4, and *T*4 and *T*5. PONV severity during *T*2 through *T*5 intervals was evaluated using the Rhodes index [15]. It described the severity of PONV, using a numerical scale from 0 to 32, including subjective (the degree of severity) and objective (with/without nausea, retching, and vomiting and times of nausea, retching, and vomiting) points of PONV. Metoclopramide (10 mg) was administered intravenously as the first-line antiemetic on demand. If metoclopramide was ineffective, then 4 mg ondansetron was administered intravenously as the second-line antiemetic on demand. Dexamethasone (5 mg) intravenously followed as the third-line antiemetic on demand.

### 2.4. Statistics

Based on a pilot study of 10 patients undergoing gynaecological laparoscopic surgery under the C group regimen, ΔMBP of 31 ± 12 mmHg, ΔHR of 28 ± 10 beats·min^−1^, and VAS at *T*2 of 51 ± 13 were obtained. The primary outcome was VAS at *T*2, and a minimum 30% VAS decrease between the groups was considered clinically significant. A sample size of 17 was calculated at 0.9 power and 0.05 *α* value. The secondary outcome was postextubation haemodynamic stability, expressed as ΔMBP and ΔHR. A minimum 30% decrease in ΔMBP and ΔHR between the groups was considered clinically significant. Sample sizes of 39 for ΔMBP and of 33 for ΔHR were calculated at a 0.9 power and a 0.05 *α* value.

Data were analysed using the Statistical Package for the Social Sciences (SPSS) version 18.0 software. The *χ* ^2^ test or Fisher's exact test was used to compare categorical variables. Student's *t*-test or the Mann-Whitney rank-sum test was used to compare the intergroup differences. The intragroup differences were analysed using the analysis of the variance on ranks for repeated measurements. All data are expressed in terms of number of patients or mean ± standard deviation. A value of *P* < 0.05 was considered statistically significant.

## 3. Results and Discussion

**Figure 1:**
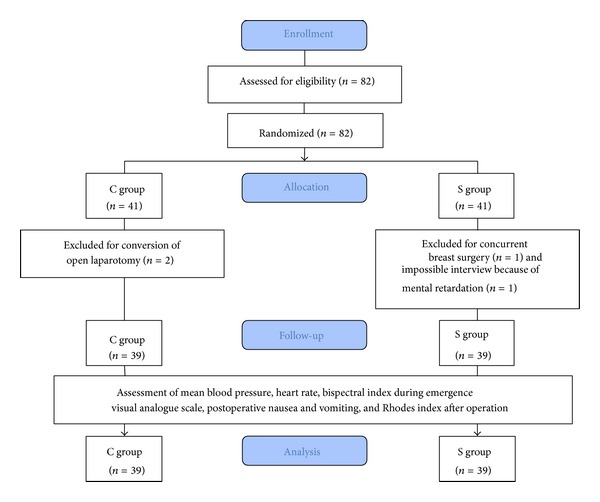
CONSORT flow diagram for the study.

In total, 82 patients were eligible and 4 patients were excluded: 2 patients in the C group were excluded for conversion to open laparotomy, 1 patient in the S group was receiving concurrent breast surgery, and 1 patient in the S group was unable to participate in the interview because of mental retardation. Thus, 39 patients in each group were included in the final analysis (Figure 1). Patient demographics and recovery times were similar between the two groups (Table 1).

The target plasma concentration of sufentanil (0.15 ng·mL^−1^) and the target tissue concentration of sufentanil (0.14 ± 0.01 ng·mL^−1^) at *T*_*e*_ were confirmed in S group. In total, 17 ± 10 *μ*g of sufentanil was administered to the S group. The infused durations of sufentanil in S group and normal saline in C group were 42 ± 24 min and 49 ± 29 min, respectively, with no significant differences noted.

**Figure 2:**
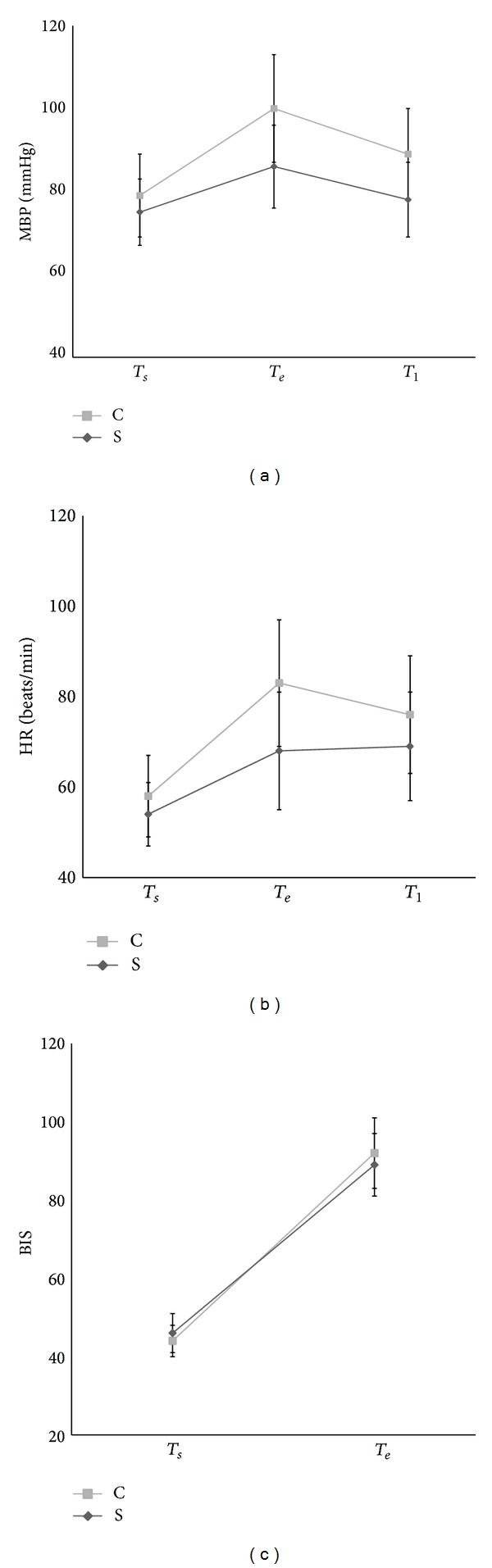
The haemodynamic and neurologic changes during emergence from anaesthesia. (a) Mean systemic blood pressure (MBP), (b) heart rate (HR), and (c) bispectral index (BIS). Abbreviations: *T*_*s*_, at initiation of sufentanil (S group) or normal saline (C group) administration; *T*_*e*_, after extubation; and *T*1, on arrival to the postanaesthetic care unit.

MBP and HR at *T*_*e*_ in the S group were significantly lower than in the C group (MBP: 86 ± 10 mmHg in S group versus 100 ± 13 mmHg in C group; *P* < 0.001) (HR: 68 ± 13 beats·min^−1^ in S group versus 83 ± 14 beats·min^−1^ in C group; *P* < 0.001) (Table 2). ΔMBP and ΔHR associated with ΔBIS were significantly lower in the S group than the C group (ΔMBP: 10 ± 9 mmHg in S group versus 20 ± 11 mmHg in C group; *P* < 0.001) (ΔHR: 14 ± 12 beats·min^−1^ in S group versus 25 ± 12 beats·min^−1^ in C group; *P* < 0.001) (ΔBIS: 43 ± 9 in S group versus 48 ± 10 in C group; *P* = 0.023) (Figure 2). Phenylephrine, ephedrine, and atropine were not administered during sufentanil or normal saline administrations (Table 2). MBP and HR at *T*1 in the S group were also significantly lower than in the C group (MBP: 78 ± 9 mmHg in S group versus 89 ± 11 mmHg in C group; *P* < 0.001) (HR: 69 ± 12 beats·min^−1^ in S group versus 76 ± 13 beats·min^−1^ in C group; *P* = 0.015) (Table 2).

Postoperative VAS peaked at *T*2 and decreased over time in the two groups. The VAS at *T*1, *T*2, and *T*3 was significantly lower in the S group than in the C group (*T*1: 21 ± 11 in S group versus 48 ± 9 in C group; *P* < 0.001) (*T*2: 27 ± 10 in S group versus 50 ± 8 in C group; *P* < 0.001) (*T*3: 19 ± 8 in S group versus 35 ± 8 in C group; *P* < 0.001) (Table 3). On-demand analgesia was not required at any time in the S group, but 13 patients at *T*1 and 7 patients at *T*2 in the C group required the first-line analgesia, ketorolac (Table 3). Second-line analgesia was not required in either group. PONV incidence and severity and the Rhodes index over time were similar between the groups, with no significant differences noted except at the *T*1 PONV (Table 3). The S group had a significantly lower PONV scale at *T*1. Neither group required the second-line or the third-line antiemetic medications.

The present study showed that sufentanil administration prior to end of remifentanil-based anaesthesia improved postoperative hyperalgesia and haemodynamic stability at extubation without delaying recovery or increasing PONV during laparoscopic gynaecological surgery.

To prevent postoperative hyperalgesia in remifentanil-based anaesthesia, longer acting opioids are commonly administered before anaesthetic emergence [6]. However, this protocol presents problems, such as delayed recovery and postoperative respiratory depression [16, 17]. Haemodynamic instability is frequently encountered during emergence from remifentanil-based anaesthesia [18]. The instability is caused by an increased sympathetic tone combined with the rapid offset of remifentanil effect [19]. As a result, several methods to prevent sympathetic tone increase are employed during emergence from anaesthesia [7, 20]. However, these methods are not capable of blunting sympathetic tone while simultaneously relieving postoperative hyperalgesia [21–23]. Drugs targeting the central nervous system are not robust enough to prevent sympathetic surge, and instead these agents contribute to the delayed recovery and postoperative respiratory depression similar to longer acting opioids [7].

Sufentanil administration prior to anaesthetic conclusion in the present study had a satisfactory effect on both postoperative hyperalgesia and haemodynamic stability. Sufentanil was not administered as single injection but instead continuously with TCI, which meticulously titrates the drug effect compared to single injection and manual infusion [24]. The target sufentanil concentration of 0.15 ng·mL^−1^ was used in the present study. This concentration is the steady-state plasma concentration associated with adequate spontaneous ventilation in 50% of patients [25]. Therefore, the risk of respiratory depression associated with longer acting opioids or centrally acting drugs was avoided. The context sensitive sufentanil half-life increased as the administration duration increased [26]. Therefore, surgical specimen procurement was the designated sufentanil start time in order to reduce the administration duration while still achieving the target concentration. Sufentanil was administered for 42 ± 24 min in the present study. Not exceeding 1 h of drug administration presumably avoids a delayed recovery.

The present study showed that the postoperative pain improved not only during the PACU stay, but also 24 h after PACU discharge even under a short duration not exceeding 1 h. However, the minimum effective plasma concentration providing postoperative analgesia (MEAC) of sufentanil is 0.025–0.050 ng·mL^−1^ [25]. Therefore, the effect of sufentanil on postoperative analgesia under the present protocol would be maintained for a long time without affecting PACU hospitalization. Bailey et al. showed that sufentanil increased the pain threshold and its duration, irrespective of the dose [27]. The effect of sufentanil on pain threshold was similarly attributed to the improved postoperative pain 24 h after PACU discharge in the present study, although there was no significant difference in use of on-demand analgesia during the *T*2-*T*3 interval in either group. The S group patients did not require additional analgesia at any time. In contrast, the use of the on-demand analgesia peaked in C group 30 min after arrival to PACU; the VAS similarly peaked at 30 min in the C group. The postoperative hyperalgesic effect of remifentanil peaked at 30 min after PACU arrival and gradually decreased thereafter; on-demand analgesia use in C group decreased during *T*2-*T*3 and, thus, showed no significant difference between the two groups.

No remarkable PONV differences were observed associated with the sufentanil in the present study, except on arrival to PACU. Potentially, the 0.15-ng·mL^−1^ sufentanil dose was unable to induce PONV, yet it was still capable of blunting the emetic centre. The significantly lower ΔBIS in the S group indicated an incomplete recovery of consciousness, although, ultimately, the BIS did not significantly affect extubation in the two groups. The lightly sedated state could influence emetic centre activity, resulting in the significantly lowered PONV on PACU arrival. Lee et al. reported that sufentanil administrated at 0.2 and 0.3 *μ*g·kg^−1^·h^−1^ before extubation suppressed cough at extubation and may thus decrease stimulation of the emetic centre [10]. In the present study, cough at extubation was not evaluated. Notably, cough at extubation is associated with increased sympathetic tone. The haemodynamic stability at extubation and on PACU arrival in the present study may potentially decrease the incidence of cough and lessen PONV through the addition of another medication like sufentanil. The total sufentanil dose administered was 22 ± 32 *μ*g, which corresponds to the dosage conducted by Lee et al. As time progressed after PACU arrival, the effect of sufentanil dissipated, and the PCA, which contains fentanyl, produces an identical impact on PONV in both groups.

There was a remaining consideration. A higher sufentanil concentration was associated with improved postoperative hyperalgesia and haemodynamic stability. Derrode et al. reported that TCI of 0.25 ng·mL^−1^ of sufentanil targeting the tissue under Gepts' model was more effective at controlling postoperative pain without compromising recovery in patients undergoing open colorectal surgery, compared to TCI of 1 ng·mL^−1^ of remifentanil targeting the tissue [28]. They also revealed that the mean plasma sufentanil concentration was 0.089 ± 0.038 ng·mL^−1^ targeting a 0.25 ng·mL^−1^ tissue concentration, using Gepts' model [28]. Namely, Gepts' model overestimated sufentanil concentration. The present study also used Gepts' model for sufentanil TCI, and the mean plasma sufentanil concentration was more likely to be lower than the target concentration. Therefore, the present study may have shown better outcomes during anaesthetic emergence and the postoperative period if the higher sufentanil concentration based on Gepts' model had been targeted.

## 4. Conclusions

Sufentanil administration before concluding remifentanil anaesthesia improved postoperative hyperalgesia and achieved haemodynamic stability at extubation without delaying recovery or increasing PONV during laparoscopic gynaecological surgery.

## Figures and Tables

**Table 1:** Patient demographics.

	C group (*N* = 39)	S group (*N* = 39)	*P*
Age (years)	40 ± 11	40 ± 13	0.802
Height (cm)	158 ± 6	160 ± 5	0.078
Weight (kg)	58 ± 9	59 ± 9	0.705
Smoking (pack × years)	0	0	—
Hx of motion sickness	1	4	0.358
Hx of PONV	0	0	—
Remifentanil (µg)	3891 ± 1581	3613 ± 976	0.352
Propofol (mg)	791 ± 375	721 ± 288	0.356
Anaesthesia time (min)	170 ± 61	156 ± 37	0.224
Surgery time (min)	137 ± 65	122 ± 36	0.210
Recovery time (min)	14 ± 4	15 ± 10	0.646
Surgical procedures			
Ovarian cystectomy	19	24	0.255
Uterine myomectomy	3	2	0.644
Vaginal hysterectomy	17	13	0.352

Data was expressed as mean ± standard deviation or number of patients.

C group: normal saline group; S group: sufentanil group; Hx: history; PONV: postoperative nausea and vomiting.

**Table 2:** Haemodynamic parameters and bispectral index.

	*T*_*s*_	*T*_*e*_	*T*1	*T*_*e*_ − *T*_*s*_
C group (*N* = 39)				
MBP (mmHg)	79 ± 10	100 ± 13	89 ± 11	20 ± 11
HR (beats·min^−1^)	58 ± 9	83 ± 14	76 ± 13	25 ± 12
BIS	44 ± 4	92 ± 9	—	48 ± 10
Medications				
Phenylephrine (µg)	—	—	—	—
Ephedrine (mg)	—	—	—	—
Atropine (mg)	—	—	—	—
S group (*N* = 39)				
MBP (mmHg)	75 ± 8	86 ± 10*	78 ± 9*	10 ± 9*
HR (beats·min^−1^)	54 ± 7	68 ± 13*	69 ± 12*	14 ± 12*
BIS	46 ± 5	89 ± 8	—	43 ± 9*
Medications				
Phenylephrine (µg)	—	—	—	—
Ephedrine (mg)	—	—	—	—
Atropine (mg)	—	—	—	—

Data is expressed as mean ± standard deviation.

C group: normal saline group; S group: sufentanil group; *T*_*s*_: initiation of sufentanil (S group) or normal saline (C group) administration; *T*_*e*_: after extubation; *T*1: on arrival at postanaesthetic care unit.

*P < 0.05 compared to the C group.

**Table 3:** Postoperative pain based on visual analogue scale (VAS) and postoperative nausea and vomiting (PONV).

	C group (*N* = 39)	S group (*N* = 39)	*P*
*T*1			
VAS	48 ± 9	21 ± 11	0.000
PONV incidence	6	1	0.108
PONV scale	0.2 ± 0.6	0.0 ± 0.2	0.048
Analgesic	13	0	0.000
Antiemetic	3	0	0.240
*T*1-*T*2			
VAS	50 ± 8	27 ± 10	0.000
PONV incidence	4	5	1.000
PONV scale	0.2 ± 0.5	0.2 ± 0.6	0.712
Analgesic	7	0	0.012
Antiemetic	2	2	1.000
*T*2-*T*3			
VAS	35 ± 8	19 ± 8	0.000
PONV incidence	15	12	0.475
PONV scale	0.6 ± 1.1	0.6 ± 0.9	0.719
Analgesic	3	0	0.240
Antiemetic	3	2	1.000
Rhodes index	3.4 ± 5.3	2.9 ± 5.4	0.613
*T*3-*T*4			
VAS	24 ± 8	24 ± 6	0.743
PONV incidence	3	2	1.000
PONV scale	0.1 ± 0.4	0.1 ± 0.2	0.629
Analgesic	0	0	—
Antiemetic	1	0	1.000
Rhodes index	0.5 ± 2.0	0.2 ± 0.9	0.471
*T*4-*T*5			
VAS	16 ± 6	15 ± 4	0.606
PONV incidence	2	0	0.494
PONV scale	0.1 ± 0.4	0.0 ± 0.0	0.155
Analgesic	0	0	—
Antiemetic	0	0	—
Rhodes index	0.3 ± 1.2	0.00 ± 0.00	0.155

Data was expressed as mean ± standard deviation or number of patients.

C group: normal saline group; S group: sufentanil group; *T*1: on arrival to the postanaesthetic care unit (PACU); *T*2: at 30 min after PACU arrival; *T*3: at 24 h after PACU discharge; *T*4: at 48 h after PACU discharge; *T*5: at 72 h after PACU discharge; PONV assessed on a three- point ordinal scale (0 = none, 1 = nausea, 2 = retching, and 3 = vomiting).
